# Female sex as an independent prognostic factor in the development of oral mucositis during autologous peripheral stem cell transplantation

**DOI:** 10.1038/s41598-020-72592-5

**Published:** 2020-09-28

**Authors:** Enikő Gebri, Attila Kiss, Ferenc Tóth, Tibor Hortobágyi

**Affiliations:** 1 Department of Dentoalveolar Surgery and Dental Outpatient Care, Faculty of Dentistry, University of Debrecen, Debrecen, Hungary; 2 Department of Haematopoietic Transplantation Centre, Faculty of Medicine, University of Debrecen, Debrecen, Hungary; 3 Department of Biomaterials and Prosthetic Dentistry, Faculty of Dentistry, University of Debrecen, Debrecen, Hungary; 4 MTA-DE Cerebrovascular and Neurodegenerative Research Group, Department of Neurology, Faculty of Medicine, University of Debrecen, Debrecen, Hungary; 5 Faculty of Medicine, Institute of Pathology, University of Szeged, Állomás utca 2, Szeged, 6725 Hungary; 6 Department of Old Age Psychiatry, Institute of Psychiatry Psychology and Neuroscience, King’s College London, London, UK; 7 Centre for Age-Related Medicine, SESAM, Stavanger University Hospital, Stavanger, Norway

**Keywords:** Lymphoma, Myeloma, Oral medicine

## Abstract

Oral mucositis (OM) is a frequent complication of stem cell transplantation-associated toxicity in haematological malignancies, contributing to mortality. Therapy still remains mainly supportive. We assessed risk factors in retrospective analysis of 192 autologous peripheral stem cell transplantation patients with lymphoma and multiple myeloma (MM), respectively. Futhermore, we examined the hormone levels both in serum and saliva during transplantation in 7 postmenopausal female patients with lymphoma compared to healthy controls using electrochemiluminescence immunoassay (ECLIA). Multivariable analysis revealed neutrophil engraftment (*p* < 0.001; *p* = 0.021) and female sex (*p* = 0.023; *p* = 0.038) as independent predictive factors in the combined patient group and in the lymphoma group, and neutrophil engraftment (*p* = 0.008) in the MM group. Of the 85 female participants 19 were pre- and 66 postmenopausal. Fifteen of the pre-, and 49 of the postmenopausal women developed ulcerative mucositis (*p* = 0.769), more often with lymphoma than MM (*p* = 0.009). Serum estrogen decreased significantly both in postmenopausal controls and transplantated patients compared to premenopausals, with no difference in saliva. Serum progesterone level was significantly (*p* = 0.026) elevated at day + 7 of transplantation, while salivary progesterone increased at day + 7 and + 14. Our results indicate a predominantly negative effect of female sex hormones on oral immunity with role in the aetiopathogenesis of OM.

## Introduction

There is a rise in incidence of malignant haematological diseases. Autologous and allogeneic haematopoietic stem cell transplantations (HSCT) are used with increasing frequency and efficiency as a therapy^1^. High-dose intensive cytostatic treatment and in some cases total body irradiation (TBI) is employed as part of the transplantation process (myeloablative conditioning therapy). Oral mucositis (OM) is a common and severe complication, graded 0–4 according to the World Health Organization (WHO)^2^. Anticancer treatment-related mucosal damage affects not only the oral cavity but also the entire gastrointestinal tract (GI), therefore it is more appropriate to use the umbrella term of mucosal barrier injury (MBI). OM causing severe pain and extensive ulcers decreases quality of life and due to damage the protective mucosal barrier results in the dissemination of oral pathogens, leading to sepsis, which is often fatal in protracted neutropenia. Prolonged hospital stay increases the risk of nosocomial infections with high mortality and strain on the health care system^3^. OM has several patient and anti-tumoural therapy related risk factors, such as nutritional deficiency, poor dental hygiene, previous exposure to prolonged immunosuppressive therapy and genetic variations affecting the pharmacodynamics of cytostatics^4^. Excretion of chemotherapeutic drugs in the saliva is locally cytotoxic^2,5^. Decreased salivary flow rate and altered saliva composition may enhance OM risk and severity^6^. Despite strenuous efforts in research and therapy, OM treatment remains mainly supportive and palliative with basic oral care, pain control, feeding and prevention of infections^7^. However, there are no predictive markers regarding the severity of OM in HSCT. Our goal was to assess and identify novel risk factors which could predict the severity of OM in haematological patients undergoing autologous peripheral stem cell transplantation (APSCT).

## Materials and methods

### Retrospective study population and design

In our study we conducted a restrospective analysis of 192 patients over a period of 4 years who had required and undergone APSCT due to malignant haematological disorder in the Haematopoietic Transplantation Centre of the Clinical Centre of the University of Debrecen, Hungary. The study was approved by the Regional Istitutional Research Ethics Committee, Clinical Center, University of Debrecen (Ethical licence: DE RKEB/IKEB 4948-2018). Diagnoses were obtained from the institutional electronic clinical database eMedSolution (T-Systems Inc. Budapest, Hungary) in accordance with the ethical approval. Written informed consents for inclusion, intraoral examination, blood and saliva sampling and research participation were obtained from all subjects and data were anonymised. No organs/tissues were procured from prisoners. All methods were carried out in accordance with relevant guidelines and regulations. The study was conducted in accordance with the Declaration of Helsinki. Eight patients were excluded due to non-compliance mainly related to disease severity.

Two large patient groups were created, lymphoma (Hodgkin (HL) and non-Hodgkin’s lymphoma (NHL)) and multiple myeloma (MM). Regarding stage at disease onset all patients were early and advanced, respectively, while regarding stage prior to the transplantation we established two groups: complete remission (CR) and very good partial remission (VGPR) were merged as a single group, whereas partial remission (PR) represented a separate group. Response categories were determined in accordance with the International Myeloma Working group (IMWG) uniform response criteria^8^. VGPR was defined as follows: serum and urine M-protein detectable by immunofixation but not on electrophoresis, or: 90% reduction in serum M-protein plus reduction in 24 h urinary M-protein by > 90% or to < 100 mg/24 h. In partial remission more than 50% reduction of serum M-protein can be observed, and reduction in 24 h urinary M-protein by ≥ 90% or to < 200 mg/24 h, and 50% reduction in the size of any plasmacytomas present at baseline. In Hodgkin lymphoma we categorised the conditioning treatments applied during the transplantation into four groups (1. BEAM, 2. R-BEAM, 3. R-BEAM-Adcetris, and 4. other). In NHL, in group 1 R-BEAM and in group 2 Z-BEAM conditioning regimen was used, whereas group 3 represented cases with any other conditioning regimen. In MM conditioning was administration of 140 mg/m^2^ melphalan in 12 patients and 200 mg/m^2^ in the rest of the patients. Treatment regimens according to the European Society for Blood and Marrow Transplantation (EBMT) recommendation^9,10^ are summarized in Supplementary Tables S1–S4. None of the patients received cryotherapy. After transplantation all patients received G-CSF with the following antimicrobial prohylaxis: (1) antibacterial prophylaxis: Leflokin (levofloxacin) 500 mg, (2) antifungal prophylaxis: Mycosyst (fluconazole) 100 mg and (3) antiviral prophylaxis: Herpesin (acyclovir) 400 mg. OM was classified according to the World Health Organization (WHO) guidelines (Grade 0–4) and the most severe appearence defined the stage in the individual patient (0 none; 1 soreness ± erythema; 2 erythema, ulcers, and patient can swallow solid food; 3 ulcers with extensive erythema and patient cannot swallow solid food; 4 mucositis to the extent that alimentation is not possible)^2^.

For the statistical analysis there were two separate groups, (1) non-ulcerative (OM0-1) and (2) ulcerative (OM2-4) mucositis^11^. Each participant had a check-up for focal dental infections and removal of infection foci prior to the transplantation. During transplantation, patients rinsed their mouths with 0.2% chlorhexidine-digluconate (CHX) solution and performed atraumatic mechanical plaque control using a special dento-brush (DenTips MDS096502, Medline Industries. Inc., Mundelein, IL, USA) after every meal during cytopenia. The time required for neutrophil engraftment was calculated as number of days with < 0.5 Giga (10^9^) per Litre (G/L) absolute neutrophil count (ANC), and for thrombocyte engraftment as number of days with < 20 G/L thrombocyte (THR) count.

We analysed the relationship between oral mucositis developed during transplantation and the following continuous variables: age at time of transplantation, time elapsed between diagnosis and transplantation (DG-TX time/month), amount of stem cells administered (10^6^/body mass kg), stem cell viability (%), number of viable cells (10^6^/body mass kg) and mononuclear cells (MNC) (10^8^/body weight kg), engraftment time (ANC < 0.5 G/L, THR < 20 G/L-days) and LDH (U/L). Of the categorical variables we analysed the relationship between oral mucositis and sex; stage of the disease at diagnosis (early *versus* advanced) and prior to transplantation (PR, VGPR, CR); the type of conditioning applied; outcome (dead or alive); infectious complications in the early post-transplantation stage (positive haemoculture); and correlation with disease subtype, where applicable. VGPR and CR, the stages immediately preceding transplantation, were conflated according to standard practice. Neutrophil engraftment is defined by peripheral absolute neutrophil count of ≥ 0.5 G/L, whereas thrombocyte engraftment by ≥ 20 G/L thombocytes, in three consecutive days^12^. Thus in our study we characterised the time of engraftment with the ˂ 0.5 G/L ANC and ˂ 20 G/L THR days, respectively.

Based on their presumed hormonal status, the 85 female patients of the study were separated into premenopausal (≤ 50 years) (n = 19) and postmenopausal (≥ 51 years) (n = 66) groups, respectively, according to published criteria^13^. All premenopausal APSCT women were on hormone replacement therapy (HRT) (5–10 mg norethisterone-acetate on cycle days 3–27, prior to and after the day of the transplantation, as long as cytopenia persisted in order to reduce the risk of severe haemorrhagic complications).

### Prospective study population and design

Estradiol (E2) and progesterone (P4) levels in serum and saliva were determined in respective healthy controls (7 pre- and 7 postmenopausal women) and in 7 postmenopausal patients with NHL undergoing APSCT, at four stages of transplantation (day − 7/− 3, 0, + 7, + 14). Sampling was performed in the Haematopoietic Transplantation Centre of the Clinical Centre of the University of Debrecen, Hungary in an 8-week period ending December 15th, 2018.

In regarding this measurement study design was aligned with STROBE recommendations and using sample size calculator Sampsize (epiGenesys, Sheffield, UK) this part of the study was a pilot. Average age was 66.71 ± 5.85 years in the postmenopausal (A), 25.43 ± 1.62 years in the premenopausal (C) control group while 54.42 ± 12.29 years in the transplantated group. Three patients were in complete morphological remission (CMR) and 4 in partial remission (PR) prior to transplantation.

### Collection of serum samples

Blood and saliva samplings were performed at the same timepoint on specified days of the peritransplantation period (on the day of hospital admission (day − 3/− 7), day of transplantation (day 0), days + 7 and + 14). Blood samples were collected using a clot activator containing serum tubes (BD, Franklin Lakes, NJ, USA), centrifuged at 1,200 × *g* for 30 min and the serum fractions were stored at − 70 °C until further processing.

### Collection of unstimulated whole saliva (UWS) samples

Sample collection was based on the protocols described previously^14,15^. In brief, following oral cavity rinse with 25 ml of physiological saline solution (B. Braun Melsungen AG, Melsungen, Germany) for 30 s, saliva was collected for 5 min in 15 ml disposable, pre-disinfected tubes (Sigma-Aldrich, St. Louis, MO, USA). Participants adapted to the condition for 5 min. Taking into account the diurnal variation of saliva constituents, samplings were done every morning at the same time, 1 h after eating, drinking or toothbrushing. Patients in the Haematopoietic Stem Cell Transplantation Centre’s steril rooms used a steril disposable oral swab (DenTips MDS096502, Medline Industries.Inc., Mundelein, IL, USA) impregnated with physiological saline solution to maintain oral hygiene during the period of cytopenia. Protease Inhibitor (Sigma-Aldrich, St. Louis, MO, USA) was added to the collected saliva samples and after aliquoting into 1.5 ml Eppendorf tubes, samples were stored at − 70 °C until processing.

### Detection of salivary and blood sample E2 and P4 levels

Saliva and serum samples stored at − 70 °C were thawed at room temperature and centrifuged at 4 °C for 10 min at 3,000 rpm. Serum (500 µl) and diluted saliva supernatant (150 µl in 450 µl Hanks’ Balanced Salt solution (Sigma-Aldrich, St. Louis, MO, USA) were filtered through 70 µm EASYstrainer cell sieve (Greiner Bio-One, Frickenhausen, Germany). Hormone levels were determined using electrochemiluminescence immunoassay (ECLIA) (Roche, Basel, Switzerland).

### Statistical analysis

Statistical analysis was performed using IBM SPSS22 software (IBM, Armonk, NY, USA). Kolmogorov–Smirnov test was used to investigate the normal distribution of data. In case of normal distribution, we compared the two groups using independent sample *t*-test in the continuous variables, whereas in non-normal distribution we applied Mann–Whitney and Wilcoxon tests. For distribution of categorical variables *Chi*-square test and Fischer exact test were used. Overall survival (OS) was calculated from the time of diagnosis to the last follow-up visit or death. Survival data were analyzed using the Kaplan–Meier method with log-rank test. Odds Ratios (OR) were obtained using binary logistic regression models. *p* < 0.05 was considered significant.

## Results

### Retrospective analysis of primary outcome of patients who underwent APSCT

The reason for HSCT was lymphoma (NHL-HL) in 85 patients and MM in 107 patients. Mean age of the 107 male patients was 54.87 ± 11.64 years (17–72 years) and of the 85 female patients was 56.13 ± 11.38 years (24–71 years). There was no significant difference between NHL-HL and MM groups regarding age and sex. The OM grade varied from grade 1 (n = 4), 2 (n = 9), 3 (n = 7) to 4 (n = 4) in HL; from grade 1 (n = 35); 2 (n = 37), 3 (n = 20) to 4 (n = 8) in MM; from grade 1 (n = 14), 2 (n = 19), 3(n = 18) to 4 (n = 7) in NHL. Considering all patients irrespective of underlying disease (n = 192), 10 did not have OM (grade 0), 53 developed grade 1, 65 had grade 2, 45 had grade 3, and 19 had grade 4 OM. In the combined lymphoma group (NHL-HL) 18 patients showed grade 1, 28 grade 2, 25 grade 3, and 11 grade 4 OM (Supplementary Fig. S1).

In the all patient group continuous variables DG-TX time (pre-treatment time) (*p* = 0.022), neutrophil engraftment (*p* < 0.001), thrombocyte engraftment (*p* < 0.001) and categorical variable female sex (*p* = 0.033) showed a significant correlation with more severe OM. There was a positive non-significant association between OM2-4 and higher LDH value (*p* = 0.07) and haemoculture positivity (*p* = 0.092). In the lymphoma group the continuous variables neutrophil engraftment (*p* = 0.005) and thrombocyte engraftment (*p* = 0.005) and the categorical variable female sex (*p* = 0.008) showed a significant correlation with OM2-4. In the myeloma group, the neutrophil engraftment (*p* = 0.011) and immunoglobulin G (IgG) (*p* = 0.049) subgroup of MM showed a significant correlation with OM2-4. The other examined variables showed no correlation with the development of oral mucositis occurring during APSCT (Table 1).

**Table 1:** Association of variables with oral mucositis in the different patient groups.

	Total patients (n = 192)			Lymphoma (n = 85)			Myeloma multiplex (n = 107)		
	OM2-4 (n = 129)	OM0-1 (n = 63)	*p*	OM2-4 (n = 64)	OM0-1 (n = 21)	*p*	OM2-4 (n = 65)	OM0-1 (n = 42)	*p*
	Mean ± SD			Mean ± SD			Mean ± SD		
Age at the time of Tx (years)	54.91 ± 12.10	56.39 ± 10.267	0.590	49.58 ± 14.17	51.90 ± 12.88	0.517	59.67 ± 6.745	58.68 ± 7.869	0.589
Dg-Tx time interval (months)	19.42 ± 25.25	16.68 ± 28.055	**0.022**	28.42 ± 31.77	29.52 ± 40.83	0.393	9.75 ± 8.701	10.10 ± 15.360	0.351
Amount of stem cells (10^6^/body mass kg)	6.43 ± 1.99	6.561 ± 2.066	0.617	6.58 ± 2.15	6.49 ± 2.30	0.829	6.263 ± 1.819	6.599 ± 1.963	0.331
Viability (%)	91.54 ± 4.91	91.028 ± 4.609	0.222	90.67 ± 5.16	90.21 ± 6.23	0.578	92.455 ± 4.51	91.408 ± 3.663	0.110
Amount of viable cells (10^6^/body mass kg)	5.85 ± 1.79	5.965 ± 1.871	0.606	5.94 ± 1.97	5.84 ± 2.04	0.931	5.755 ± 1.588	6.020 ± 1.813	0.403
MNC (10^8^/body mass kg)	4.29 ± 3.54	3.964 ± 2.592	0.826	4.86 ± 4.30	4.40 ± 2.43	0.828	3.671 ± 2.375	3.759 ± 2.671	0.978
ANC ˂ 0.5 G/L (days)	7.17 ± 2.38	5.62 ± 1.679	**< 0.001**	8.11 ± 2.16	6.62 ± 1.46	**0.005**	6.25 ± 2.236	5.12 ± 1.565	**0.011**
THR ˂ 20 G/L (days)	8.33 ± 5.01	5.07 ± 3.104	**< 0.001**	10.16 ± 4.70	7.62 ± 2.76	**0.005**	6.52 ± 4.667	4.74 ± 2.829	0.132
LDH (U/L)	248.45 ± 107.77	218.607 ± 68.58	*0.070*	273.37 ± 130.71	230.05 ± 58.36	0.305	221.709 ± 67.44	212.25 ± 73.654	0.279

	n (%)		*p*	n (%)		*p*	n (%)		*p*
Male	65 (50%)	42 (67%)	**0.033**	34 (53%)	18 (86%) 3 (14%)	**0.008**	31 (48%)	24 (57%)	0.340
Female	64 (50%)	21 (33%)		30 (47%)			34 (52%)	18 (43%)	
**Stage at the time of the diagnosis**									
I–II	19 (46%)	15 (56%)		4 (20%)			15 (71%)	14 (74%)	0.873
II–III	22 (54%)	12 (44%)	0.457	16 (80%)			6 (29%)	5 (26%)	
**Pretransplantational stage**									
I–II	70 (59%)	35 (62%)	0.689	38 (64%)			32 (54%)	26 (68%)	0.164
II–III	48 (41%)	21 (38%)		21 (36%)			27 (46%)	12 (32%)	
**Conditional therapy**									
1	31 (51%)	10 (48%)							
2	10 (17%)	5 (24%)							
3	15 (25%)	6 (28%)							
4	4 (7%)	0							
**Last status**									
1	35 (27%)	10 (16%)	0.084	18 (28%)			17 (26%)	6 (14%)	0.144
0	94 (73%)	53 (84%)		46 (72%)			48 (74%)	36 (86%)	
**Infection**									
1	37 (29%)	11 (17%)	*0.092*	23 (36%)			14 (22%)	4 (10%)	0.105
0	92 (71%)	52 (83%)		41 (64%)			51 (78%)	38 (90%)	
**IgA/IgG**									
1	–	–	–	–			9 (17%)	11 (37%)	**0.049**
2	–	–		–			43 (83)	19 (63%)	

*p* < 0.05 was considered significant. *p* values with bold are significant, while with italic, are non-significant, but close to it.

Statistical analysis: independent samples *t*-test, Mann–Whitney test, *Chi*-square test and Fisher’s exact test were used.

*ANC* absolute neutrophil count, *Dg* diagnosis, *IgA* immunoglobulin A, *IgG* immunoglobulin G, *LDH* lactate-dehydrogenase,* n* numero, *OM* oral mucositis, *THR* thrombocyte count, *Tx* transplantation.

Univariable analysis revealed in the all patient group in terms of OM2-4 the following prognostic factors: neutrophil engraftment (OR 1.461, 95% CI 1.225–1.744, *p* < 0.001), thrombocyte engraftment (OR 1.178, 95% CI 1.078–1.288, *p* < 0.001), female sex (OR 1.969, 95% CI 1.052–3.687, *p* = 0.034) and lymphoma group (OR 1.969, 95% CI 1.052–3.678, *p* = 0.034). Increased LDH values (OR 1.004, 95% CI 1–1.009, *p* = 0.067) and cases with positive haemoculture (OR 1.901, 95% CI 0.894–1.041, *p* = 0.095) may represent higher risks in the development of more severe OM. In the lymphoma subgroup, in addition to neutrophil engraftment (OR 1.584, 95% CI 1.134–2.214, *p* = 0.007), thrombocyte engraftment (OR 1.279, 95% CI 1.043–1.57, *p* = 0.018) and female sex (OR 5.294, 95% CI 1.418–19.762, *p* = 0.013), absence of B symptoms (OR 7.000, 95% CI 1.085–45.16, *p* = 0.041) turned out to be prognostic factors of OM2-4. Hodgkin and non-Hodgkin’s lymphoma subgroups were combined due to the small number of cases in the HL group. Absence of B symptoms by itself did not appear as a prognostic factor among patients with NHL. In the myeloma group, neutrophil engraftment (OR 1.39, 95% CI 1.09–1.773, *p* = 0.008) and thrombocyte engraftment (OR 1.128, 95% CI 1.009–1.262, *p* = 0.034) were prognostic factors while the IgG subgroup (OR 2.766, 95% CI 0.984–7.773, *p* = 0.054) suggests a higher risk for the development of OM.

Multivariable analysis revealed that in the all patient group and in the lymphoma group neutrophil engraftment (OR 1.492, 95% CI 1.228–1.813, *p* < 0.001; OR 1.476, 95% CI 1.061–2.052, *p* = 0.021) and female sex (OR 2.301, 95% CI 1.124–4.714, *p* = 0.023; OR 4.190, 95% CI 1.081–16.240; *p* = 0.038) could be considered independent predictive factors (Classification ratio: 67.7%, Nagelkerke coefficient: 0.172); (Classification ratio: 78.8%, Nagelkerke coefficient: 0.236) in the development of OM2-4. In the myeloma group neutrophil engraftment (OR 1.39, 95% CI 1.09–1.773, *p* = 0.008) (Classification ratio: 62.6%, Nagelkerke coefficient: 0.105) appeared as an independent prognostic factor (Table 2).

**Table 2:** Risk factors of oral mucositis.

	Total patients		Lymphoma		Myeloma multiplex	
	OR (95% CI)	*p*	OR (95% CI)	*p*	OR (95% CI)	*p*
**Univariable analysis**						
Age at the time of Tx (years)	0.988 (0.964–1.015)	0.371	0.988 (0.952–1.025)	0.526	1.024 (0.970–1.082)	0.384
Dg-Tx time interval (months)	1.004 (0.991–1.016)	0.542	0.999 (0.985–1.013)	0.897	0.999 (0.970–1.029)	0.950
Amount of stem cells (10^6^/body mass kg)	0.967 (0.830–1.127)	0.668	1.021 (0.81–1.285)	0.863	0.908 (0.731–1.127)	0.382
Viability (%)	1.022 (0.959–1.090)	0.503	1.016 (0.925–1.116)	0.744	1.062 (0.963–1.172)	0.226
Amount of viable cells (10^6^/body mass kg)	0.965 (0.813–1.146)	0.688	1.025 (0.787–1.335)	0.853	0.909 (0.715–1.157)	0.439
MNC (10^8^/body mass kg)	1.033 (0.930–1.147)	0.547	1.034 (0.89–1.2)	0.664	0.989 (0.833–1.166)	0.868
ANC < 0.5 G/L (days)	1.461 (1.225–1.744)	**< 0.001**	1.584 (1.134–2.214)	**0.007**	1.39 (1.09–1.773)	**0.008**
THR < 20 G/L (days)	1.178 (1.078–1.288)	**< 0.001**	1.279 (1.043–1.57)	**0.018**	1.128 (1.009–1.262)	**0.034**
LDH (U/L)	1.004 (1–1.009)	*0.067*	1.005 (0.998–1.012)	0.168	1.002 (0.996–1.008)	0.526
Female versus male	1.969 (1.052–3.687)	**0.034**	5.294 (1.418–19.762)	**0.013**	1.462 (0.67–3.194)	0.340
Advanced stage at the time of the diagnosis	1.447 (0.545–3.842)	0.458	0.571 (0.054–6.079)	0.643	1.120 (0.278–4.508)	0.873
Advanced stage before transplantation	1.143 (0.594–2.198)	0.689	0.553 (0.19–1.606)	0.276	1.828 (0.778–4.297)	0.166
Infection	1.901 (0.894–1.041)	*0.095*	1.122 (0.396–3.178)	0.828	2.608 (0.795–8.554)	0.114
HL-NHL	1.455 (0.468–4.524)	0.518	1.455 (0.468–4.524)	0.518	–	–
MM-NHL	0.563 (0.282–1.124)	0.103	–	–	–	–
L-MM	1.969 (1.052–3.687)	**0.034**	–	–	–	–
Stage of missing of B symptoms	–	–	7.000 (1.085–45.16)	**0.041**		
IgA versus IgG	–	–	–	–	2.766 (0.984–7.773)	**0.054**
Kappa versus lambda	–	–	–	–	0.644 (0.291–1.425)	0.277
Dosis of melphalan (≥ 200 mg/m^2^)	–	–	–	–	1.046 (0.301–3.629)	0.944
MM pretreatment	–	–	–	–	1.559 (0.464–5.231)	0.472
**Multivariable analysis**						
ANC ˂ 0.5 G/L (days)	1.492 (1.228–1.813)	**< 0.001**	1.476 (1.061–2.052)	**0.021**	1.39 (1.09–1.773)	**0.008**
Gender	2.301 (1.124–4.714)	**0.023**	4.190 (1.081–16.240)	**0.038**		

*p* < 0.05 was considered significant. *p* values with bold are significant, while with italic, are non-significant, but close to it.

Statistical analysis: binary logistic regression was used.

*ANC* absolute neutrophil count, *Dg* diagnosis, *HL* Hodgkin lymphoma, *IgA* immunoglobulin A, *IgG* immunoglobulin G, *L* lymphoma, *LDH* lactate-dehydrogenase, *MM* Multiple myeloma, *MNC* mononuclear cells, *n* numero, *NHL* Non-Hodgkin’s lymphoma*, OM* oral mucositis, *THR* thrombocyte count, *Tx* transplantation.

**Figure 1:**
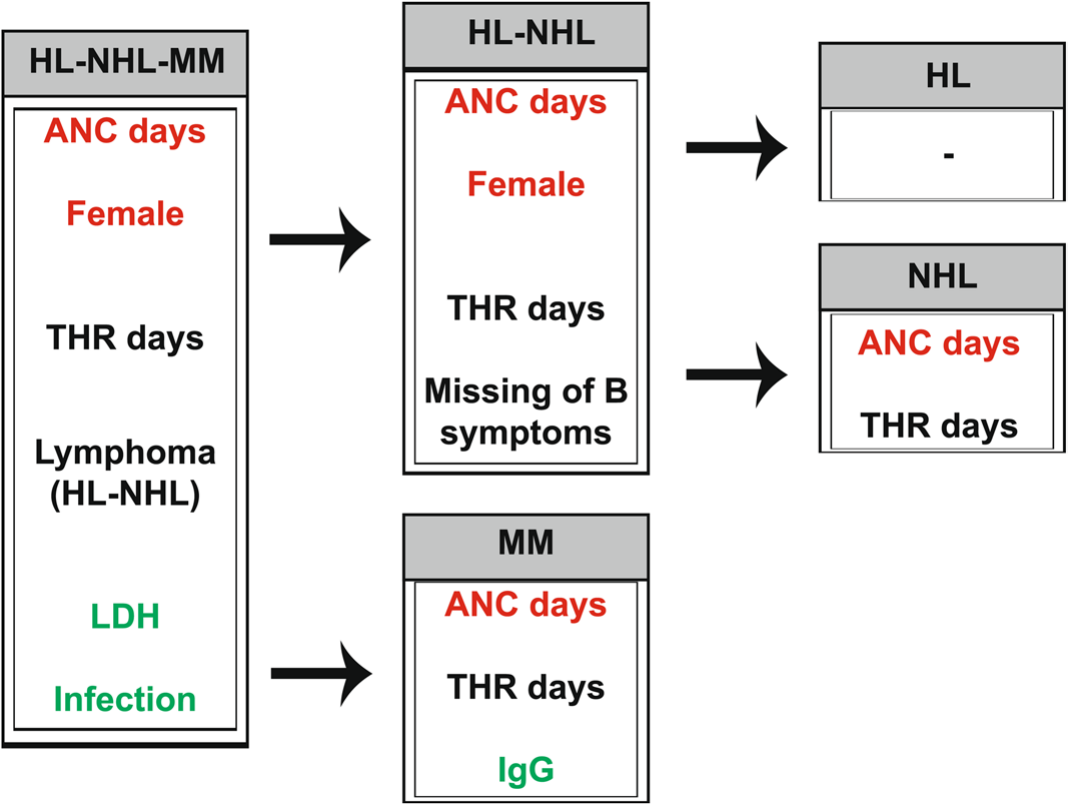
Risk factors and independent predictive factors of oral mucositis. *ANC* absolute neutrophil count, *HL* Hodgkin lymphoma, *IgG* immunoglobulin G, *LDH* lactate-dehydrogenase, *NHL* Non-Hodgkin’s lymphoma, *THR* thrombocyte count. Flowchart summary of statistical tests’ results. Colour codes/Letter markings: Red: independent prognostic factors revealed by multivariable analysis Black: prognostic factors revealed by multivariable analysis Green: higher risk in terms of ulcerative mucositis by univariable analysis.

Time to neutrophil engraftment (7.774 ± 2.10 days) was significantly (*p* < 0.001) longer in the lymphoma group than in the MM (5.80 ± 2.07 days). At the same time, neutrophil engraftment was the strongest predictive factor in the NHL group (OR 1.598, 95% CI 1.101–2.321, *p* = 0.014); (thrombocyte engraftment: OR 1.239, 95% CI 1.004–1.529, *p* = 0.046; female sex: OR 5.320, 95% CI 1.077–26.276, *p* = 0.040) (Fig. 1).

### Secondary outcomes of retrospective analysis of women who underwent APSCT

As next, since female sex has been shown to have a major role in the development of OM2-4, we conducted further analysis.

The 85 female patients were classified into two groups based on their hormonal status^13^. Patients ≤ 50 years were grouped as premenopausal and those ≥ 51 years as postmenopausal, as we mentioned earlier. Based on this classificiation 19 women were premenopausal and 66 postmenopausal. Of the 19, 15 (78.95%), and of the 66, 49 (74.24%) patients developed ulcerative mucositis (OM2-4). There was no significant difference between the two groups (*p* = 0.771).

We did our calculations for the total patient group and the individual patient group, respectively, and got similar results. In the lymphoma subgroup 16 (88.89%) out of 18 premenopausal women, while 14 (93.33%) out of 15 postmenopausal women developed severe OM. The difference was not significant here, either (*p* = 1). In the myeloma group, 33 out of 49 postmenopausal female patients developed ulcerative OM while the corresponding ratio in the premenopausal group was 1 out of 3 (33.33%). No significant difference was found here, either (*p* = 0.114).

However, ulcerative mucositis was significantly (*p* = 0.009) more frequent in the lymphoma group than in the MM group (30 of the 33 patients with lymphoma-90.9%, while 34 out of the 53 patients with MM-65.38%, *p* = 0.009).

### Overall survival with and without ulcerative mucositis

**Figure 2:**
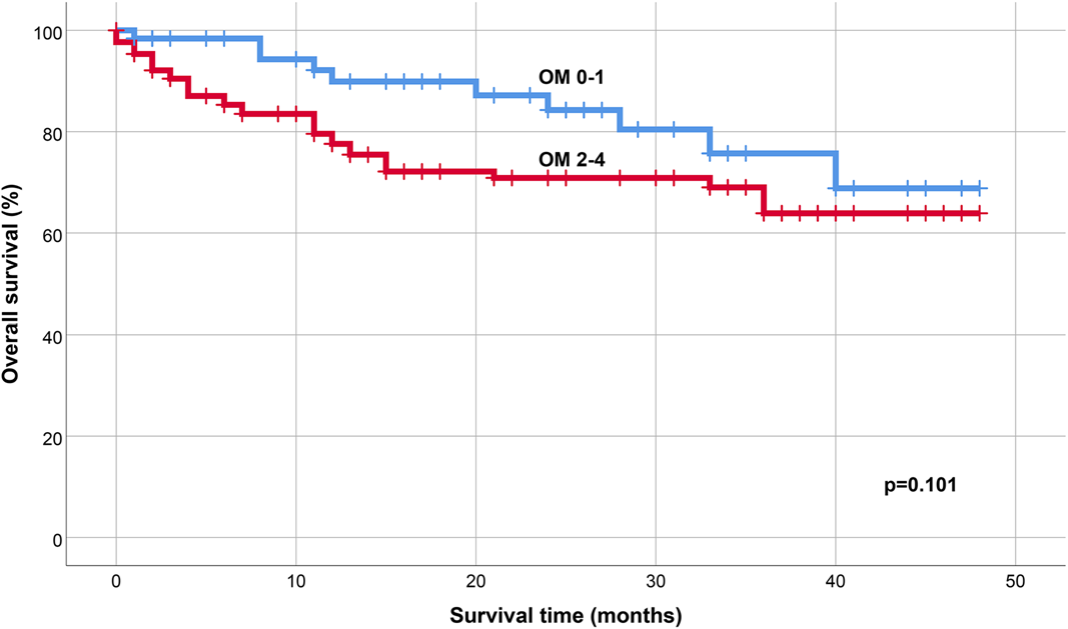
Kaplan–Meier overall survival curves of patient groups. Analysis of overall survival (OS) with (OM2-4) and without (OM0-1) ulcerative mucositis.

Correlation was assessed between average post-transplantational survival time and ulcerative mucositis. OS was 5.17 months shorter in the combined patient group (HL, NHL, MM) if ulcerative mucositis developed (OM2-4: 35.34 months (31.83–38.85); OM0-1: 40.51 months (26.42–44.61) (*p* = 0.101) (Fig. 2).

### Determination of serum and salivary E2 and P4 levels

A physiological decrease was observed in serum E2 level in the postmenopausal control group (A) compared to the premenopausal (C) (*p* = 0.004), while there was no significant difference in salivary E2 level (*p* = 0.069). Both in serum and saliva, P4 levels were significantly decreased in the postmenopausal controls compared to the premenopausal group (*p* = 0.017, *p* = 0.004).

**Figure 3:**
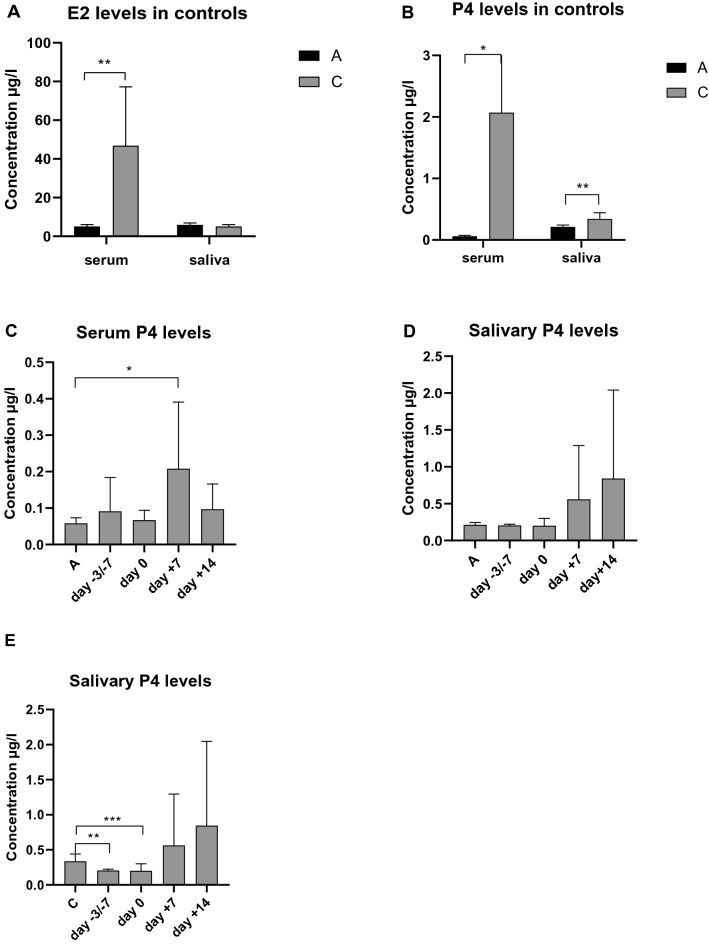
Saliva and serum levels of estrogen (E2) and progesterone (P4) in the pre- and postmenopausal control groups and during APSCT (autologous peripheral stem cell transplantation). (**A**) Serum and salivary E2 levels in the control groups. Serum E2 decreased significantly (*p* = 0.004) in the postmenopausal group compared to the premenopausal group. There was no significant difference (*p* = 0.069) in salivary E2 levels. (**B**) Serum and salivary P4 levels in the control groups. Both in serum and saliva P4 levels were significantly decreased in the postmenopausals compared to the premenopausals (*p* = 0.017, *p* = 0.004). (**C**) Serum P4 levels in the postmenopausal control group compared to the transplantated patients. Significant difference was observed at day + 7 (0.026). (**D**) Salivary P4 levels in the postmenopausal control group and in the transplantated group. A tendency for increase was observed at days + 7 and + 14. (**E**) Salivary P4 levels in the premenopausal control group and in the transplantated patients. Physiological significant decreased was observed in remission (day − 3/ − 7) and day 0, and a tendency for elevation at days + 7 and + 14 compared to the premenopausal controls. On the X axis C is for premenopausal control group and A is for postmenopausal control group. Values are expressed as sample means; error bars represent the estimates of standard deviations calculated from three parallel measurements (*p < 0.05, **p < 0.01, ***p < 0.001). Statistical analysis: Kolmogorov–Smirnov test, Mann–Whitney test, Wilcoxon test.

Serum P4 was more elevated in the transplantated patients compared to the postmenopausal controls at all four stages of transplantation, at day + 7 significantly (*p* = 0.026). Salivary P4 was higher, although not significantly at days + 7 and + 14 compared to the two other stages of APSCT and to controls (≥ 51), respectively. Although decrease in salivary P4 level was significant in the postmenopausal controls compared to the premenopausals, a tendency for increase was observed in postmenopausal APSCT patients at day + 7 and day + 14 compared not only to the postmenopausal controls, but also to the premenopausals (Fig. 3).

Serum E2 decreased significantly (*p* = 0.004) in the patient group compared to the premenopausal controls, while there was no significant difference between E2 serum and salivary hormone levels of postmenopausal controls and patients with NHL undergoing APSCT in the postmenopause. In summary, we didn’t find any significant changes of E2 in relation to NHL and/or due to the APSCT in the patient group.

### Unstimulated whole saliva (UWS) in controls and in patients during APSCT

**Figure 4:**
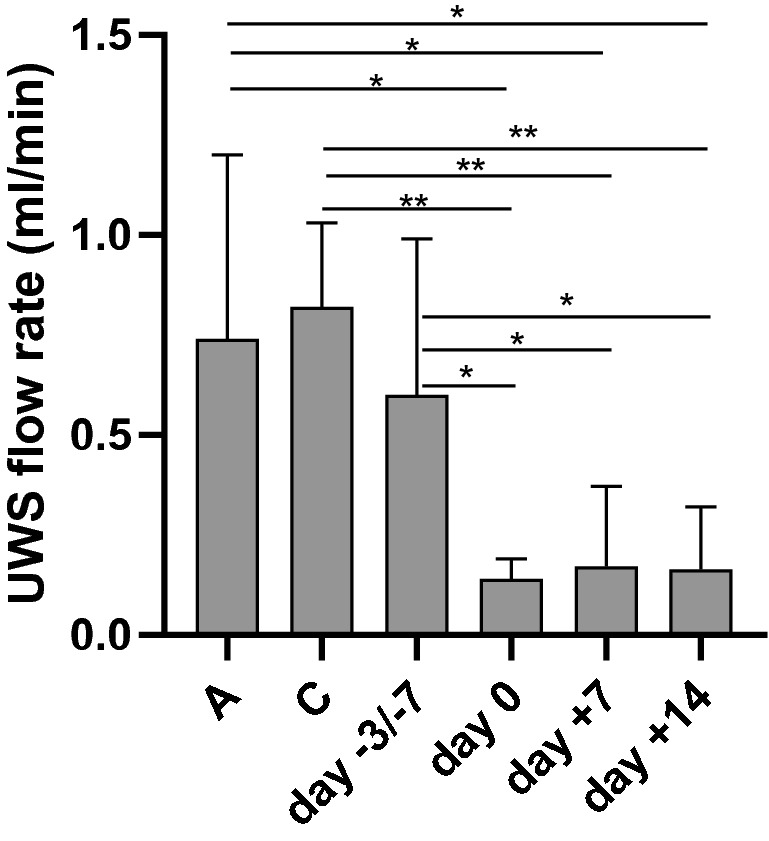
Salivary flow rate of unstimulated whole saliva (UWS) in controls and in patients during autologous peripheral stem cell transplantation (APSCT). On the X axis C is for premenopausal control group and A is for postmenopausal control group. Values are expressed as sample means; error bars represent the estimates of standard deviations calculated from three parallel measurements (*p < 0.05, **p < 0.01, ***p < 0.001). Statistical analysis: Kolmogorov–Smirnov test, Mann–Whitney test and Wilcoxon test**.**

There was no significant difference (*p* = 0.628) in UWS flow rate between the pre- and postmenopausal control groups. During APSCT, significant decrease was observed at day 0, day + 7 and day + 14 in UWS flow rate between the pre- and postmenopausal control groups (*p* = 0.004, *p* = 0.004, *p* = 0.004); (*p* = 0.048, *p* = 0.030, *p* = 0.018), and between the day of admission of APSCT (day − 3/− 7) (*p* = 0.043, *p* = 0.043, *p* = 0.043), respectively (Fig. 4)*.* There was a significant positive correlation (*p* = 0.008, r = 0.928) between serum E2 level and UWS flow rate in the premenopausal group.

## Discussion

OM is associated with increased mortality in haematological malignancies. Numerous studies have attempted to prevent it or reduce its severity, with limited success^7^. There is a new wave in OM studies with focus on risk factors implicated in other diseases of the oral cavity including cancer. Our results indicate that neutrophil engraftment is an independent predictive factor of OM both in lymphoma and MM whereas female sex predisposes to ulcerative OM in lymphoma only.

### Serum LDH levels

Pre-transplantation serum LDH levels are a good reflection of the remaining tumour mass, thus its prognostic role is important. Huang et al*.* examined the prognostic value of pre-radiotherapy serum LDH levels in patients with non-metastatic oropharyngeal carcinoma (OPC)^16^. Their results showed that increased LDH levels in HPV+ OPC patients were an independent prognostic factor in terms of survival. The results of our own study suggest that with marginally increased pre-transplantational serum LDH levels a higher prevalence of ulcerative oral mucositis can be predicted, which supports its negative prognostic role.

### Neutrophil engraftment and oral mucositis

Neutrophils in the first lines of defense play a crucial role in protection againts infections and promote wound healing^17^. Platelets contribute to tissue remodelling after injury and inflammation^18^. Timely insertion and proliferation of stem cells in neutrophil and thrombocyte engraftments are essential in reducing risk and severity of OM. Earlier studies have reported at length on the relationship between neutrophil engraftment and OM, and the positive correlation has also been confirmed between the duration of mucositis and neutrophil engraftment^19^. Reduced as opposed to standard dose MTX in allogeneic HSCT patients resulted in shorter neutrophil engraftment time and faster recovery from mucositis^19^. The anti-inflammatory, antioxidant and wound-healing promoting effects of erythropoietin containing mouthwash in patients who underwent APSCT, reducing severity and incidence of OM without significant effect on blood neutrophil and thrombocyte engraftment^12^, further supports the role of local factors in the aetiology and therapy of OM. Several studies have unanimously concluded that oral engraftment is an earlier sign of adherence and recovery of stem cells than blood engraftment. Furthermore, it is a more sensitive indicator of oral mucositis and reflects the status of local immunity^20–22^.

### Female sex

The predictive role of the female sex regarding OM in APSCT has been confirmed by earlier studies^23,24^, however, no in-depth analysis like our study has been reported. The dose of chemotherapy applied over a larger body surface area (BSA) per body mass in women may explain the sex difference^25^. Furthermore, women have increased risk of cytotoxicity and oral mucositis possibly partially due to hormone-related post-translational modifications^26^. We confirmed that APSCT-related OM was more frequent and severe in women than in men. Severe ulcerative OM (OM2-4) was more frequent in lymphoma than in myeloma, pontentially due to the longer pre-treatment time. There was no significant difference in incidence of ulcerative OM between pre- and postmenopauls. To explain this, we conducted hormonal analysis in the prospective part of our study. In the postmenopause E2 levels were physiologically decreased (Fig. 3A). In premenopausal patients, hormone levels were affected by progestin and norethisterone-acetate administered before and during transplantation to reduce the risk of haemorrhagic complications. However, we observed significantly increased serum progesterone levels at day + 7 of APSCT (which is the deepest point of cytopenia and the most severe grade of OM) also in postmenopausal patients with lymphoma (Fig. 3C), who also had increased incidence and severity of OM in our retrospective analysis. Therefore, we assume that the hormonal changes identified in our study (increased progesterone in postmenopausal patients in particular) do exert a strong effect on OM development. It is generally known that sex hormones have direct and indirect effects on the physiology of the oral cavity. In future studies, the effects of hormonal changes on the oral epithelium, periodontium, oral microbiome, the composition of the saliva and function of the immune system could be correlated with tissue-specific localization, quantity and functional status of sex hormone receptors.

### Oral epithelium

The adverse effect of decreased serum estrogen on the mucosa is well known: the oral epithelium becomes thin and atrophic, accompanied by a deterioration of mucosal integrity and immunity^27^, whereas estrogen supplementation and HRT improves mucosal integrity^28–30^. The oral mucosa also responds to progesterone^31^, high levels being detrimental, especially in immuno-compromised patients^32–34^. There were elevated serum and salivary progesterone levels in our patients during APSCT (Fig. 3C,D,E).

### Periodontium and oral microbiome

The increased sensitivity of periodontal tissues to sex hormones has been widely investigated. Estrogen increases epithelial keratinization, stimulates proliferation of fibroblasts and epithelial basal cells in the gingiva^27,35^. In postmenopausal women the prevalence of periodontitis is significantly higher compared with premenopausals^36^. Periodontal pathogens such as *Porphyromonas gingivalis, Tannerella forsythia*, *Prevotella intermedia* play a role in the development and progression of periodontitis, while HRT decreases the amount of this pathogen flora^37,38^. In our patients with APSCT-related cytopenia the cycle was suspended with norethisterone-acetate. This progestin decreases keratinization, favours colonisation of Gram-negative bacteria (e.g. *Bacteroides, Prevotella* sp.). Progesterone also enhances inflammatory response to local irritative factors thereby increasing the risk and severity of OM^35^. These findings support the notion that the predictive role of female sex in the development of OM during APSCT is a consequence of the shifted hormonal balance.

During HSCT, patients with chronic gingivitis or periodontitis have higher incidence of bacteriaemia and ulcerative mucositis^39^. In the preengraftment stage there is often anaerob blood stream infection (ABI) with *Fusobacterium and Porphyromonas* species of oral and upper GI tract origin, whereas during postengraftment *Bacteroides and Clostridium* species of lower GI tract origin colonize^40^. Changes in microbial diversity occure rapidly in patients with ulcerative oral mucositis during APSCT^41^. However, we did not find significant correlation between evidence of infection by haemoculture and ulcerative oral mucositis (OM2-4) in our retrospective study. Futher research is recommended with wider range of methods, such as swab culture and polymerase chain reaction (PCR).

In conclusion, hormonal changes (particularly decreased E2 and increased P4 levels) have a negative effect on the periodontium and favour the proliferation of periodonthopathogens, which play an increased role in the development of bacteriaemia and ulcerative OM during HSCT.

### Salivary glands and saliva

Saliva has a fundamental role in the homeostasis of the oral cavity, lubrication of the mucosa, and protection against microorganisms and oxidative stress. Changes in its organic and anorganic components act as a sensitive mirror of the body’s defence mechanisms. Its diagnostic importance is outstanding and comparable with the serum. Steroid hormone levels in the saliva usually correlate with the active hormone levels in the serum^42,43^. Our salivary P4 results correlated well with serum P4 levels both in healthy controls and in the transplantated patients (Fig. 3), whereas it did not reach the level of significance regarding salivary E2. Salivary secretion and changes in its components are under significant sex hormone regulation and estrogen and progesterone receptors are present in the salivary glands^31,44^. Most studies report decreased salivary flow rate in postmenopause noting the positive effect of HRT^45,46^, while others enhance the decrease of stimulated whole saliva only^47,48^. We found no significant difference in UWS flow rate between the pre-and postmenopausal controls, and a positive correlation between serum E2 level and UWS flow rate in the premenopausals (Fig. 4). Several studies have demonstrated decreased salivary flow rate during APSCT under the influence of conditioning treatment^6^, in concert with our results (Fig. 4).

There is impairment of salivary antimicrobial defence and mucosal integrity during APSCT^6,49^. Thus, we can conclude that postmenopausal hormonal changes increase the risk of OM that occurs during APSCT.

### Oral immunity

Although the interactions involved are not yet completely understood, the interrelationship between production of sex hormones and the functioning of the immune system has been confirmed in many studies^35^. This is also supported by increased incidence of autoimmune diseases among women^35^. Estrogen has a stimulating effect on the immune system, as opposed to progesterone and androgens^50^. E2 regulates glycosylation of IgG and its role in immunological processes^51^. Also, salivary secretory immunoglobulin A (sIgA) levels change parallel to serum E2 levels^52^ and altered N-glycosylation of salivary sIgA and serum IgA is a possible biomarker in oral mucositis^15^. Importantly, high-dose progesterone attenuates the antibacterial activity of neutrophils^53^, whereas decreased estrogen aggravates dento-alveolar infections^54^ and impairs leukocyte chemotaxis^35^. These observations further underline the importance of sex hormones in OM and their relevance to APSCT. Their role in PMN chemotaxis may be crucial in ulcerative OM development and support our finding that neutrophil engraftment is an independent prognostic factor of OM.

## Conclusion

Hormonal changes in women play a fundamental role in the aetiopathogenesis of OM during APSCT. These changes may exert their complex effect directly or indirectly on components of saliva and on oral epithelium, microbiome and immunity. Decreased estrogen levels adversely affect several aspects of oral homeostasis, and elevated progesterone levels both in serum and saliva may play a role in the weakening of the mucosal barriers not only in the pre- but also in postmenopause in particular during APSCT. Monitoring serum progesterone levels in women undergoing APSCT may be a suitable tool in the assesment of mucosal immunity, function and risk of severe OM.

## Supplementary information

Supplementary Information.

## Data Availability

The data sets used for analysis for this study are available from the corresponding author upon reasonable request.

## Abbreviations

ABI: Anaerobic bloodstream infection
ANC: Absolute neutrophil count
APSCT: Autologous peripheral stem cell transplantation
BEAM: BCNU, etoposide, cytosine arabinoside, melphalan
BMI: Body mass index
CHX: Chlorhexidine digluconate
CI: Confidence interval
CMR: Complete morphological remission
CR: Complete remission
DG: Diagnosis
E2: 17-beta-estradiol
GI: Gastrointestinal tract
G-CSF: Granulocyte-colony stimulating factor
G/L: Giga per Litre
GVHD: Graft-versus-host disease
HL: Hodgkin lymphoma
HSCT: Haematopoietic stem cell transplantation
HRT: Hormone replacement therapy
IgA: Immunoglobulin A
IgG: Immunoglobulin G
IMWG: International Myeloma Working Group
LDH: Lactate-dehydrogenase
MTX: Methotrexate
MBI: Mucosal barrier injury
MM: Multiple myeloma
NHL: Non-Hodgkin lymphoma
OM: Oral mucositis
OM0-1: Non-ulcerative mucositis
OM2-4: Ulcerative mucositis
OR: Odds ratios
OPC: Oropharyngeal carcinoma
OS: Overall survival
P4: Progesterone
PCR: Polymerase chain reaction
PR: Partial remission
PMN: Polymorphonuclear leukocyte
R-BEAM: Rituximab-BCNU, etoposide, cytosine arabinoside, melphalan
sIgA: Secretory immunoglobulin A
TBI: Total body irradiation
THR: Thrombocyte
TX: Transplantation
UWS: Unstimulated whole saliva
VGPR: Very good partial remission
WHO: World Health Organization
Z-BEAM: Zevalin-BCNU, etoposide, cytosine arabinoside, melphalan

## References

[1] Kanate AS (2020). Indications for hematopoietic cell transplantation and immune effector cell therapy: guidelines from the American Society for Transplantation and Cellular Therapy. Biol. Blood Marrow Transplant..

[2] Niscola P (2007). Mucositis in patients with hematologic malignancies: an overview. Haematologica.

[3] Blijlevens NMA, Logan RM, Netea MG (2009). Mucositis: from febrile neutropenia to febrile mucositis. J. Antimicrob. Chemother..

[4] Salavaggione OE, Wang L, Wiepert M, Yee VC, Weinshilboum RM (2005). Thiopurine S-methyltransferase pharmacogenetics: variant allele functional and comparative genomics. Pharmacogenet. Genom..

[5] Sonis ST (2004). Perspectives on cancer therapy-induced mucosal injury. Cancer.

[6] van Leeuwen S (2018). Early salivary changes in multiple myeloma patients undergoing autologous HSCT. Oral Dis..

[7] Jensen SB, Peterson DE (2014). Oral mucosal injury caused by cancer therapies: current management and new frontiers in research. J. Oral Pathol. Med..

[8] Durie B (2006). International uniform response criteria for multiple myeloma. Leukemia.

[9] Storti F (2018). Melphalan 140 mg/m2 or 200 mg/m2 for autologous transplantation in myeloma: results from the Collaboration to Collect Autologous Transplant Outcomes in Lymphoma and Myeloma (CALM) study. A report by the EBMT Chronic Malignancies Working Party. Haematologica.

[10] Gyurkocza B, Sandmaier BM (2014). Conditioning regimens for hematopoietic cell transplantation: one size does not fit all. Blood.

[11] Chaudhry HM (2016). The incidence and severity of oral mucositis among allogeneic hematopoietic stem cell transplantation patients: a systematic review. Biol. Blood Marrow. Transplant..

[12] Hosseinjani H (2017). The efficacy of erythropoietin mouthwash in prevention of oral mucositis in patients undergoing autologous hematopoietic SCT: a double-blind, randomized, placebocontrolled trial. Hematol. Oncol..

[13] McKinlay SM, Brambilla DJ, Posner JG (1992). The normal menopause transition. Am. J. Hum. Biol..

[14] Meszaros B (2020). N-glycomic analysis of Z(IgA1) partitioned serum and salivary immunoglobulin A by capillary electrophoresis. Curr. Mol. Med..

[15] Gebri E (2020). N-glycosylation alteration of serum and salivary immunoglobulin A is a possible biomarker in oral mucositis. J. Clin. Med..

[16] Huang SH (2016). Independent adverse prognosis of elevated serum LDH in human papillomavirus-related oropharyngeal cancer. Int. J. Radiat. Oncol. Biol. Phys..

[17] Wang J (2018). Neutrophils in tissue injury and repair. Cell Tissue Res..

[18] Kasirer-Friede A (2018). The platelet response to tissue injury. Front. Med..

[19] Matsukawa T (2016). Reduced-dose methotrexate in combination with tacrolimus was associated with rapid engraftment and recovery from oral mucositis without affecting the incidence of GVHD. Int. J. Hematol..

[20] Akpek G, Knight RD, Wright DG (2003). Use of oral mucosal neutrophil counts to detect the onset and resolution of profound neutropenia following high-dose myelosuppressive chemotherapy. Am. J. Hematol..

[21] Cheretakis C, Dror Y, Glogauer M (2005). A noninvasive oral rinse assay to monitor engraftment, neutrophil tissue delivery and susceptibility to infection following HSCT in pediatric patients. Bone Marrow Transplant..

[22] Pink R (2009). Salivary neutrophils level as an indicator of bone marrow engraftment. Biomed. Pap. Med. Fac. Univ. Palacky Olomouc Czech Repub..

[23] Vokurka S (2006). Higher incidence of chemotherapy induced oral mucositis in females: a supplement of multivariate analysis to a randomized multicentre study. Support. Care Cancer.

[24] Valeh M (2018). Factors affecting the incidence and severity of oral mucositis following hematopoietic stem cell transplantation. Int. J. Hematol. Stem Cell Res..

[25] Blijlevens N (2008). Prospective oral mucositis audit: oral mucositis in patients receiving high-dose melphalan or BEAM conditioning chemotherapy—European Blood and Marrow Transplantation Mucositis Advisory Group. J. Clin. Oncol..

[26] Huang RS, Kistner EO, Bleibel WK, Shukla SJ, Dolan ME (2007). Effect of population and gender on chemotherapeutic agent-induced cytotoxicity. Mol. Cancer Ther..

[27] Alzoubi EE (2017). Oral manifestations of menopause. J. Dent. Health Oral Disord. Ther..

[28] Litwack D, Kennedy JE, Zander HA (1970). Response of oral epithelia to ovariectomy and estrogen replacement. J. Periodontal Res..

[29] Tarkkila L, Furuholm J, Tiitinen A, Meurman JH (2012). Saliva in perimenopausal and early postmenopausal women. A 2-year follow-up study. Clin. Oral Investig..

[30] Croley TE, Miers C (1978). Epithelial changes in the oral mucosa resulting from a variation in hormone stimulus. J. Oral Med..

[31] Välimaa H (2004). Estrogen receptor-β is the predominant estrogen receptor subtype in human oral epithelium and salivary glands. J. Endocrinol..

[32] Solomon M, Itsekson AM, Lev-Sagie A (2013). Autoimmune progesterone dermatitis. Curr. Dermatol. Rep..

[33] Grunnet KM, Powell KS, Miller IA, Davis LS (2017). Autoimmune progesterone dermatitis manifesting as mucosal erythema multiforme in the setting of HIV infection. JAAD Case Rep..

[34] Minicucci EM (2009). Oral stomatitis induced by endogenous progesterone: case report. Gynaecol. Endocrinol..

[35] Mariotti A (1994). Sex steroid hormones and cell dynamics in the periodontium. Crit. Rev. Oral Biol. Med..

[36] Haas AN, Rösing CK, Oppermann RV, Albandar JM, Susin C (2009). Association among menopause, hormone replacement therapy, and periodontal attachment loss in Southern Brazilian Women. J. Periodontol..

[37] Brennan RM, Genco RJ, Hovey KM, Trevisan M, Wactawski-Wende J (2007). Clinical attachment loss, systemic bone density, and subgingival calculus in postmenopausal women. J. Periodontol..

[38] Tarkkila L, Kari K, Furuholm J, Tiitinen A, Meurman JH (2010). Periodontal disease-associated micro-organisms in peri-menopausal and post-menopausal women using or not using hormone replacement therapy. A two-year follow-up study. BMC Oral Health.

[39] Raber-Durlacher JE (2013). Periodontal status and bacteremia with oral viridans streptococci and coagulase negative staphylococci in allogeneic hematopoietic stem cell transplantation recipients: a prospective observational study. Support. Care Cancer.

[40] Okinaka K (2017). Characteristics of anaerobic bloodstream infections after allogeneic hematopoietic stem cell transplantation. J. Hematop. Cell Transplant..

[41] Laheij AMGA (2019). Microbial changes in relation to oral mucositis in autologous hematopoietic stem cell transplantation recipients. Sci. Rep..

[42] Greabu M (2009). Saliva—a diagnostic window to the body, both in health and in disease. J. Med. Life.

[43] Tivis LJ, Richardson MD, Peddi E, Arjmandi B (2005). Saliva versus serum estradiol: Implications for research studies using postmenopausal women. Prog. Neuro-Psychopharmacol. Biol. Psychiatry.

[44] Shick PC, Riordan GP, Foss RD (1995). Estrogen and progesterone receptors in salivary gland adenoid cystic carcinoma. Oral Surg. Oral Med Oral Pathol. Oral Radiol. Endod..

[45] Mahesh DR (2014). Evaluation of salivary flow rate, PH and buffer in pre, post & post menopausal women on HRT. J. Clin. Diagn. Res..

[46] Nagler RM, Hershkovich O (2005). Relationships between age, drugs, oral sensorial complaints and salivary profile. Arch. Oral Biol..

[47] Fenoll-Palomares C (2004). Débito basal, pH y capacidad tampón de la secreción salivar en sujetos sanos. Rev. Esp. Enferm. Dig..

[48] Minicucci EM, Pires RBC, Vieira RA, Miot HA, Sposto MR (2013). Assessing the impact of menopause on salivary flow and xerostomia. Aust. Dent. J..

[49] Avivi I (2009). Oral integrity and salivary profile in myeloma patients undergoing high-dose therapy followed by autologous SCT. Bone Marrow Transplant..

[50] McMurray RW (2001). Estrogen, prolactin, and autoimmunity: Actions and interactions. Int. Immunopharmacol..

[51] Ercan A (2017). Estrogens regulate glycosylation of IgG in women and men. JCI Insight.

[52] Gómez E (1993). Hormonal regulation of the secretory IgA (sIgA) system: estradiol- and progesterone-induced changes in sIgA in parotid saliva along the menstrual cycle. Am. J. Reprod. Immunol..

[53] Roth JA, Kaeberle ML, Hsu WH (1982). Effect of estradiol and progesterone on lymphocyte and neutrophil functions in steers. Infect. Immun..

[54] Youssef H, Stashenko P (2017). Interleukin-1 and estrogen protect against disseminating dentoalveolar infections. Int. J. Oral Sci..

