# Supplementary material for: Female sex as an independent prognostic factor in the development of oral mucositis during autologous peripheral stem cell transplantation
Source: Sci Rep. 2020 Sep 28;10:15898. doi: 10.1038/s41598-020-72592-5 (PMC7522228; doi:10.1038/s41598-020-72592-5)
Supplement: Supplementary file 1 — Supplementary Information. [file 41598_2020_72592_MOESM1_ESM.pdf]

Female sex as an independent prognostic factor in the development of oral mucositis during autologous peripheral stem cell transplantation

Enikő Gebri[1], Attila Kiss[2], Ferenc Tóth[3], Tibor Hortobágyi[4,5,6,7]

[1]Department of Dentoalveolar Surgery and Dental Outpatient Care, Faculty of Dentistry, University of Debrecen, Debrecen, Hungary

[2]Department of Haematopoietic Transplantation Centre, Faculty of Medicine, University of Debrecen, Debrecen, Hungary

[3]Department of Biomaterials and Prosthetic Dentistry, Faculty of Dentistry, University of Debrecen, Debrecen, Hungary

[4]MTA-DE Cerebrovascular and Neurodegenerative Research Group, Department of Neurology, Faculty of Medicine, University of Debrecen, Debrecen, Hungary

[5]Institute of Pathology, Faculty of Medicine, University of Szeged, Szeged, Hungary

[6]Department of Old Age Psychiatry, Institute of Psychiatry Psychology and Neuroscience, King's College London, London, UK

[7]Centre for Age-Related Medicine, SESAM, Stavanger University Hospital, Stavanger, Norway

Corresponding author:

Tibor Hortobágyi *MD PhD DSc FRCPath EFN*

Institute of Pathology, Faculty of Medicine,

University of Szeged

Szeged, Állomás utca 2.

H-6725, Hungary

Email: hortobagyi.tibor@med.u-szeged.hu

Supplementary Figure

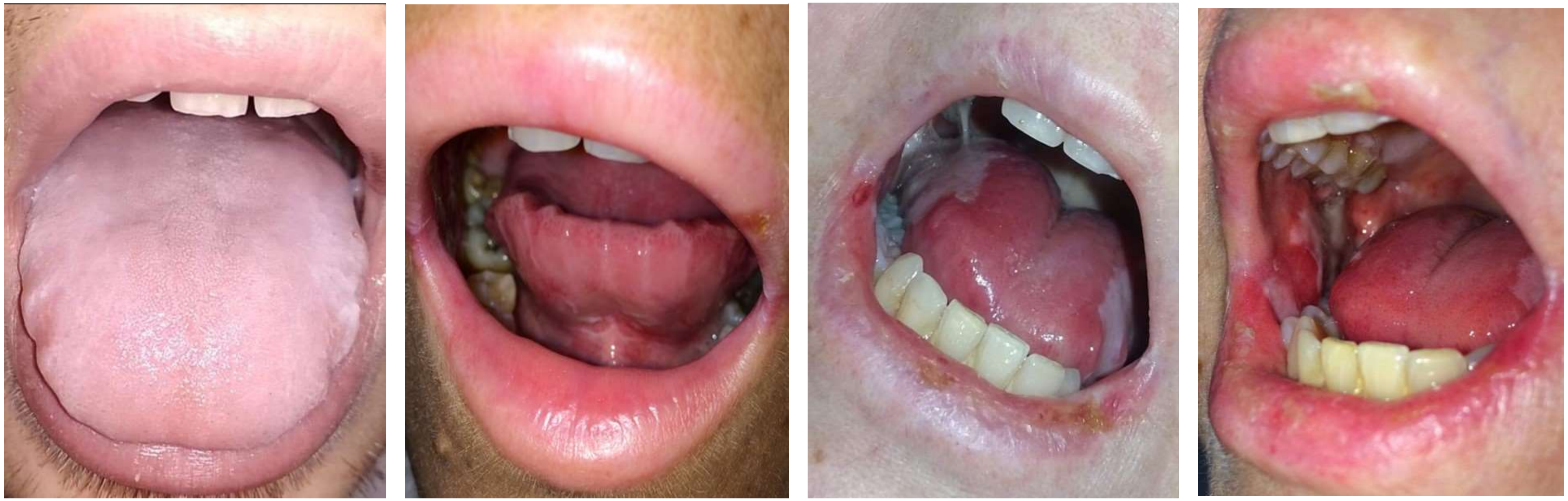

A **B** **C** **D**

Supplementary Figure S1. Representative images of oral mucositis WHO grades I-IV in our patient population. (A) Grade I. Mild oedema and anaemia, impression of teeth on the lateral borders of the tongue. Patient had soreness. **(B) Grade II.** Impression of teeth on the ventral surface of the tongue with solid mucosal erosions with erythema on the frenulum. Patient could swallow solid food. **(C) Grade III.** Ulcers with extensive erythema on the dorsal surface of the tongue with some solid scale on the lower vermillion. Patient could not swallow solid food. **(D) Grade IV.** Extensive, confluent ulcers in the whole oral cavity. Alimentation was not possible, total parenteral nutrition was necessary.

Supplementary Tables

Supplementary Table S1. Autologous conditioning protocol BEAM I-II.

BEAM		BEAM I.			BEAM II.		
Day	Agent	Dose	Vehicle	Duration of administration	Dose	Vehicle	Duration of administration
-7	Carmustine (BCNU)	300 mg/m^2	500 ml 0.9% NaCl	1 h	300 mg/m^2	500 ml 0.9% NaCl	1 h
-6	Etoposide	200 mg/m^2	500 ml 0.9% NaCl	1 h	2x100 mg/m^2	2 x 500 ml 0.9% NaCl	2 x 60 min
	Cytarabine	2×200 mg/m^2	2×100 ml 0.9% NaCl	2×3 h	2x200 mg/m^2	2×100 ml 0.9% NaCl	2 x 3 h
-5	Etoposide	200 mg/m^2	500 ml 0.9% NaCl	1 h	2x100 mg/m^2	2 x 500 ml 0.9% NaCl	2 x 60 min
	Cytarabine	2×200 mg/m^2	2×100 ml 0.9% NaCl	2×3 h	2x200 mg/m^2	2×100 ml 0.9% NaCl	2 x 3 h
-4	Etoposide	200 mg/m^2	500 ml 0.9% NaCl	1 h	2x100 mg/m^2	2×500 ml 0.9% NaCl	2 x 60 min
	Cytarabine	2×200 mg/m^2	2×100 ml 0.9% NaCl	2×3 h	2x200 mg/m^2	2×100 ml 0.9% NaCl	2 x 3 h
-3	Etoposide	200 mg/m^2	500 ml 0.9% NaCl	1 h	2x100 mg/m^2	2×500 ml 0.9% NaCl	2 x 60
	Cytarabine	2×200 mg/m^2	2×100 ml 0.9% NaCl	2×3 h	2x200 mg/m^2	2×100 ml 0.9% NaCl	2 x 3 h
-2	Melphalan	140 mg/m^2	100 ml 0.9% NaCl	0,5-1 h	140 mg/m^2	100 ml 0.9% NaCl	30-60 min

Supplementary Table S2. Autologous conditioning protocol BEAM III-IV.

BEAM		BEAM III.			BEAM IV.		
**Day**	**Agent**	**Dose**	**Vehicle**	**Duration of administration**	**Dose**	**Vehicle**	**Duration of administration**
-7	Carmustine (BCNU)	300 mg/m^2	500 ml 0.9% NaCl	1 h	300 mg/m^2	500 ml 0.9% NaCl	1 h
-6	Etoposide	200 mg/m^2	500 ml 0.9% NaCl	1 h	200 mg/m^2	500 ml 0.9% NaCl	1 h
	Cytarabine	2×200 mg/m^2	2×100 ml 0.9% NaCl	2×3 h	200 mg/m^2	100 ml 0.9% NaCl	3 h
-5	Etoposide	200 mg/m^2	500 ml 0.9% NaCl	1 h	200 mg/m^2	500 ml 0.9% NaCl	1 h
	Cytarabine	2×200 mg/m^2	2×100 ml 0.9% NaCl	2×3 h	200 mg/m^2	100 ml 0.9% NaCl	3 h
-4	Etoposide	200 mg/m^2	500 ml 0.9% NaCl	1 h	200 mg/m^2	500 ml 0.9% NaCl	1 h
	Cytarabine	2×200 mg/m^2	2×100 ml 0.9% NaCl	2×3 h	200 mg/m^2	100 ml 0.9% NaCl	3 h
-5	Etoposide	200 mg/m^2	500 ml 0.9% NaCl	1 h	200 mg/m^2	500 ml 0.9% NaCl	1 h
	Cytarabine	2×200 mg/m^2	2×100 ml 0.9% NaCl	2×3 h	200 mg/m^2	100 ml 0.9% NaCl	3 h
-2	Melphalan	140 mg/m^2	100 ml 0.9% NaCl	0,5-1 h	140 mg/m^2	100 ml 0.9% NaCl	0.5-1 h

Supplementary Table S3. Autologous conditioning protocol R-BEAM; Z-BEAM

R-BEAM					Z-BEAM				
**Day**	**Agent**	**Dose**	**Vehicle**	**Duration of administration**	**Day**	**Agent**	**Dose**	**Vehicle**	**Duration of administration**
-8	Rituximab (or day -7)	375 mg/m^2	500 ml 0.9% NaCl	3 h	-21	Rituximab	375 mg/m^2	500 ml 0.9% NaCl	3 h
-7	Carmustine (BCNU)	300 mg/m^2	500 ml 0.9% NaCl	1 h	-14	Zevalin	3,8 MBq/m^2	-	bolus
	Rituximab (or day -8)	375 mg/m^2	500 ml 0.9% NaCl	3 h	-7	Carmustine (BCNU)	300 mg/m^2	500 ml 0.9% NaCl	1 h
-6	Etoposide	200 mg/m^2	500 ml 0.9% NaCl	1h	-6	Etoposide	200 mg/m^2	500 ml 0.9% NaCl	1 h
	Cytarabine	2×200 mg/m^2	2×100 ml 0.9% NaCl	2×3 h		Cytarabine	2×200 mg/m^2	2×100 ml 0.9% NaCl	2×3 h
-5	Etoposide	200 mg/m^2	500 ml 0.9% NaCl	1 h	-5	Etoposide	200 mg/m^2	500 ml 0.9% NaCl	1 h
	Cytarabine	2×200 mg/m^2	2×100 ml 0.9% NaCl	2×3 h		Cytarabine	2×200 mg/m^2	2×100 ml 0.9% NaCl	2×3 h
-4	Etoposide	200 mg/m^2	500 ml 0.9% NaCl	1 h	-4	Etoposide	200 mg/m^2	500 ml 0.9% NaCl	1 h
	Cytarabine	2×200 mg/m^2	2×100 ml 0.9% NaCl	2×3 h		Cytarabine	2×200 mg/m^2	2×100 ml 0.9% NaCl	2×3 h
-3	Etoposide	200 mg/m^2	500 ml 0.9% NaCl	1 h	-3	Etoposide	200 mg/m^2	500 ml 0.9% NaCl	1 h
	Cytarabine	2×200 mg/m^2	2×100 ml 0.9% NaCl	2×3 h		Cytarabine	2×200 mg/m^2	2×100 ml 0.9% NaCl	2×3 h
-2	Melphalan	140 mg/m^2	100 ml 0.9% NaCl	0,5-1 h	-2	Melphalan	140 mg/m^2	100 ml 0.9% NaCl	0,5-1 h

R-BEAM-Adcetris: Adcetris completion (Brentuximab-vedotin) in case of therapy refractory or relapsed Hodgkin's lymphoma in one infusion before R-BEAM conditioning 1.8 mg/kg iv. in 30 minutes.

Supplementary Table S4. Autologous conditioning protocol R-BEAM; Z-BEAM

MELPHALAN				
**Day**	**Agent**	**Dose**	**Vehicle**	**duration of administration**
-2	melphalan	200 mg/m^2	500 ml 0.9% NaCl	max. 1h
-2	melphalan	140 mg/m^2	500 ml 0.9% NaCl	max. 1h

By individual consideration only. Primarily in multiple myeloma (MM) patients in poor clinical condition, ≥65y with 30ml/min GFR in secondary transplantation.
